# Problem drinking and physical intimate partner violence against women: evidence from a national survey in Uganda

**DOI:** 10.1186/1471-2458-12-399

**Published:** 2012-06-06

**Authors:** Nazarius Mbona Tumwesigye, Grace Bantebya Kyomuhendo, Thomas Kennedy Greenfield, Rhoda K Wanyenze

**Affiliations:** 1 Department of Epidemiology and Biostatistics, School of Public Health, Makerere University College of Health Sciences, Kampala, Uganda; 2 Department of Women studies, School of Women and Gender Studies, Makerere University College of Humanities and Social Sciences, Kampala, Uganda; 3 Alcohol Research Group, Public Health Institute, Emeryville, CA, USA; 4 Department of Disease Control and Environmental Health, School of Public Health, Makerere University College of Health Sciences, Kampala, Uganda

**Keywords:** Gender based violence, Domestic violence, Physical intimate partner violence, Drunkenness, Intoxication, Alcohol consumption, Frequency of alcohol consumption, Logistic regression, Socio-economic status, Economic empowerment, Violence against women

## Abstract

**Background:**

Problem drinking has been identified as a major risk factor for physical intimate partner violence (PIPV) in many studies. However, few studies have been carried on the subject in developing countries and even fewer have a nationwide perspective. This paper assesses the patterns and levels of PIPV against women and its association with problem drinking of their sexual partners in a nationwide survey in Uganda.

**Methods:**

The data came from the women’s dataset in the Uganda Demographic and Health Survey of 2006. Problem drinking among sexual partners was defined by women’s reports that their partner got drunk sometimes or often and served as the main independent variable while experience of PIPV by the women was the main dependent variable. In another aspect problem drinking was treated an ordinal variable with levels ranging from not drinking to getting drunk often. A woman was classified as experiencing PIPV if her partner pushed or shook her; threw something at her; slapped her; pushed her with a fist or a harmful object; kicked or dragged her, tried to strangle or burn her; threatened/attacked her with a knife/gun or other weapon. General chi-square and chi-square for trend analyses were used to assess the significance of the relationship between PIPV and problem drinking. Multivariate analysis was applied to establish the significance of the relationship of the two after controlling for key independent factors.

**Results:**

Results show that 48% of the women had experienced PIPV while 49.5% reported that their partners got drunk at least sometimes. The prevalence of both PIPV and problem drinking significantly varied by age group, education level, wealth status, and region and to a less extent by occupation, type of residence, education level and occupation of the partner. Women whose partners got drunk often were 6 times more likely to report PIPV (95% CI: 4.6-8.3) compared to those whose partners never drank alcohol. The higher the education level of the women the less the likelihood of experiencing PIPV (p_trend_ < 0.001). Similar relationship was found between wealth status and experiencing PIPV.

**Conclusions:**

Problem drinking among male partners is a strong determinant of PIPV among women in Uganda. PIPV prevention measures should address reduction of problem drinking among men. Longerterm prevention measures should address empowerment of women including ensuring higher education, employment and increased income.

## Background

Intimate partner violence (IPV) is a widespread form of gender-based violence. IPV is commonly defined as violence by one adult perpetrated on another, both parties being involved in an intimate relationship as a spouse or sexual partner [1]. IPV includes acts of physical aggression, psychological abuse, forced sexual intercourse, or any other controlling behavior [2]. Both men and women suffer from IPV but most of the cases are committed by men against women [3]. Women are more likely to be repeat victims of IPV and they are more likely to experience more severe forms of IPV than men [4]. The United Nations (UN) regards violence against women as a human rights concern and public policy issue [5] while the World Health Organization (WHO) regards it as an important public health problem [6].

IPV has a direct impact on women’s health [7,8] and child health [9], and accounts for a significant number of deaths among women. Studies from a range of countries show that 40–70% of female murder victims were killed by their husband or boyfriend [6]. Worldwide, evidence suggests that alcohol is closely associated with occurrence of IPV [2]. The major focus of this paper is problem drinking. Although this has several definitions and may be subjective, for the purpose of this paper problem drinking is defined as having gotten drunk “sometimes” or “often” versus not having gotten drunk [10]. In another aspect problem drinking is compared with not drinking alcohol.

Compared to other forms of IPV it is easier to measure physical intimate partner violence (PIPV) and compare this between countries since more work has been done with this construct compared to other forms of IPV. Another challenge with using alternative definitions of IPV, such as those that include sexual violence, is that these have more varying definitions and hence systematic comparative data across countries are less available [11]. Therefore, the concern of this paper is PIPV, the physical form of IPV. Relatively little work has been done in Uganda and in the East African region in relation to the association between alcohol consumption and PIPV against women.

Alcohol consumption reduces self control and affects cognitive and physical functioning which reduces the ability of an individual to negotiate non-violent conflict resolution [12]. Alcohol consumption has been found to increase the occurrence and severity of domestic violence [13]. In addition, alcohol consumption by partners may cause financial problems, aggressive behaviour, childcare problems and other related problems which can lead to violence against women [14-16]. A study in the USA found that 30 to 40% of the men and 27 to 34% of the women who perpetrated violence against their partners were taking alcohol at the time of the event [17]. A multi-country study in Chile, India, Egypt and the Philippines identified regular alcohol consumption by the partner as a risk factor for any life time PIPV against women across the four countries [18]. A study in the UK found that 32% of IPV related events in UK occurred when the perpetrator was under the influence of alcohol [4]. A study in India found that excessive drinking predicted partner violence (OR = 28.7; 95% CI 11.5–71.7) [19].

Detailed analysis in several studies has found that it is problem drinking that contributes most to PIPV rather than just drinking alcohol [2,19]. A study among battered women in UK found that 52% of offending males were described as frequently drunk while 22% had episodes of heavy drinking [20]. A study of injured women in the USA found that 65% of those injured by partners had partners with alcohol abuse problems [21]. Problem drinking significantly predicted perpetration of PIPV among cohabitees in a US study even after controlling for gender roles and other risk factors [22]. Thus suggesting that the higher the level of alcohol consumption the higher the likelihood of PIPV perpetrated by males towards females [23]. Another study found that among women attending emergency services the higher the level of alcohol abuse by their [14]partners, the greater the likelihood that injuries were due to IPV [21].

Apart from problem drinking, many other factors are recognized as contributing to PIPV. Some of these factors include poverty, lack of women’s empowerment [24], urban residence and poor involvement in decision making [25], young age, low education and unemployment [26] being single or divorced/separated [27]. These factors need to be considered when studying the relationship between problem drinking and PIPV.

Levels of PIPV vary widely across countries but they tend to be higher in developed than developing countries. A multi-country study commissioned by WHO and conducted from 2000 to 2003 found that the lifetime prevalence of PIPV ranged from 13% in Japan to 61% in Peru. In the same study Tanzania, which neighbours Uganda, had a PIPV prevalence of 47% [28]. In a survey of 24,000 women in Canada only 7% reported having been victims of PIPV in the previous 5 years [29]. A national study in the USA showed that 23% of the black couples, 11.5% of the white couples, and 17% of the Hispanic couples reported an event of male-to-female partner violence in the previous 12 months [17]. However, comparison of PIPV between countries has to be done cautiously because definitions of PIPV vary by country and studies [2]. The best comparison is offered in multi-country studies [2].

Uganda has high per capita alcohol consumption and high prevalence of PIPV. In 2004 Uganda had the highest per capita alcohol consumption in the world with an average of 19.4 l per capita [30]. Despite the decline in 2011, Uganda still had the second highest per capita alcohol (11.93 l) consumption in Africa and was rated 28^th^ in the world [31]. A study in 2003 found that the prevalence of alcohol consumption was 55% among men and 40% among women [32]. In the previous 12 months, 59% of male drinkers and 23% of female drinkers had taken at least 5 drinks on a single day [32]. Regarding PIPV, a study in two Ugandan districts found that 41% of women had been beaten or harmed by a partner [33].

According to the 2006 Uganda Demographic and Health Survey (UDHS), 48% of ever-married women experienced physical violence by their partners, and this proportion was much higher than that of physical violence against men (20%) [34]. Given the reported relationship between alcohol consumption and PIPV in many studies, it is important to determine the patterns and strength of the relationship in a country with high prevalence of both heavy alcohol intake among men and PIPV experienced by women. Some studies in Uganda have already attributed high frequent occurrences of PIPV to high alcohol consumption [35,36] but the evidence to date was based on small sample studies. Physical violence against men is a problem but the violence against women is more prevalent and more severe in the country, and for this reason this paper is focused on PIPV against women.

Much is known about factors associated with PIPV and alcohol consumption but relatively less is known about the association between problem drinking and PIPV in developing countries, especially in Africa. Furthermore, most of the few studies carried out in Africa have not had a nationwide perspective. This paper presents the prevalence of PIPV committed against women and the prevalence of problem drinking among their partners, as well as the association between PIPV and problem drinking.

## Methods

### Dataset

The data analyzed for this paper came from the women’s data set of the Uganda Demographic and Health Survey (UDHS) of 2006, conducted by Macro International, Inc. and the Uganda Bureau of Statistics (UBOS). The data set is openly available. The sample for the survey was drawn using a two-stage cluster sampling technique where the Primary Sampling Unit (PSU) was an Enumeration Area (EA) demarcated from the 2002 national census. In the first stage 368 PSUs were randomly selected using Probability Proportional to Size (PPS) from EAs listed by district and rural/urban residence. The same list of EAs had been used in the 2005 national household survey [37]. In the second stage 25–30 households were randomly selected from each of the EA using systematic sampling technique. The data collection was through face-to-face interviews. A more detailed description of the survey methods can be obtained from the 2006 UDHS published report [34].

The UDHS survey reached 8,531 women aged 15–49 but only 2,087 (24.5%) were randomly selected for the interview on domestic violence while 1,748 (20.5%) were asked questions specifically on violence against them by their male sexual partners (spouses or other sexual partners). Only 1,743 of the 1748 provided answers to questions on both PIPV and alcohol consumption. Computation of the prevalence of PIPV and significance testing of its variation by different background characteristics were based on weighted data. The overall eligible women response rate of the survey was 95% [34].

### Measures

PIPV referred to events when a male partner to the respondent did any of the following in previous 12 months to the respondent: pushed, shook or threw something at her, slapped, punched with fist or with something harmful that could hurt her, kicked or dragged or beat her, tried or actually choked or burnt her on purpose, threatened or attacked her with a knife/gun or other weapon [34]. While important, sexual and emotional violence are not considered in this paper. The definition of PIPV used in this study is similar to that used in the recent WHO study [38].

The UDHS survey probed alcohol consumption of each woman’s partner including whether her partner drank alcohol and, if he did, how often he got drunk. Answers to the two questions were used to create a new drinking variable with the following categories: i) does not drink ii) drinks but never gets drunk iii) gets drunk sometimes iv) gets drunk very often. For defining the prevalence of problem drinking the variable was dichotomized: ever got drunk versus never drank or drank but did not get drank.

The dependent variable was experience of PIPV by the respondent while the key independent variable was problem drinking of the male partner. Independent variables investigated for moderating effects of the relationship between PIPV and problem drinking were age group of the women, their marital status, participation in decision making at home, religion, region, type of residence (rural/urban), education level, occupation, wealth quintile, age difference with partner, education level of the partner and occupation of the partner. The regions are Central, Kampala city, Eastern, Northern and Western Uganda. The wealth quintiles were developed for all women using household assets, services, and amenities [39]. To assess women’s decision making autonomy, information was sought on women’s participation in four different types of household decisions: On the respondent’s own health care, on making major household purchases, on making household purchases for daily needs, and on visiting her family or relatives. A woman had fair autonomy or participation in decision making if she unilaterally or jointly with her partner made decisions on at least 2 of the 4 items. Her participation in decision making was regarded as low if she did not make any unilateral or joint decision with her partner on any of the four items or the decision was made on only one item. The age group of the women was categorised in 5 year age groups while the difference in age with their partners was grouped as i) partner same age or younger ii) 1–4 years older iii) 5–9 years older iv) 10+ older. The 5 year age group categorisation was meant to allow for ease of comparison with previous studies.

### Analysis strategy

Data analysis was carried out using STATA V10 and it started with general description of the sample in terms of socio-demographic characteristics. The prevalence of problem drinking was computed using the general sampling weights while that of PIPV was computed using special partner violence weights. Both weights were provided in the data set. The chi-square test of significance was applied to assess the significance of the variation in the proportions.

The next stage of analysis involved multivariate logistic regression of the relationship between PIPV and problem drinking. Here the four level problem drinking variable was used in order to assess the effects of each level of problem drinking on PIPV. Both unadjusted and adjusted odds ratios for being a victim of PIPV are presented together with their 95% confidence intervals. Multivariate backward elimination criteria was applied with inclusion criteria of p < =0.1 for variables from the bivariate analysis. The p-value of 0.1 is midway between the more inclusive criteria of 0.2 and less inclusive criteria of 0.05. Variables were excluded from the models depending on the contribution to goodness of fit of the models [39] and Wald’s test p-value [40]. The potential for the listed independent variables to modify the relationship between problem drinking and PIPV was tested by adding interaction terms one at a time and testing their significance using the Wald’s test.

## Results

### Characteristics of the women

Most of the women were of young or middle age with 69% (1,198) under 35 years. Twenty three percent (408) had not attained any formal education while only 14%(250) had attained secondary education (Table 1). Eighty five percent (1,487) of the women lived in rural areas. Eighty four percent (1,465) were either married or living together with their partners. Women’s level of participation in decision making at home was fair as 70% (1021) had either unilaterally or jointly made 2 to 4 decisions with their partners on issues concerning health care, large household purchases, purchases for daily needs and visiting family and relatives. Each of the four geographical regions of the country contributed substantially to the sample but the central region which includes Kampala city contributed highest (28%) number of women. Most of the women (77%) were Christians. Sixty eight percent (1,181) of the women were engaged in agriculture or were self employed. Regarding economic status the women were categorized by wealth quintile. The medium wealth quintile had the smallest number of women (331) while the second poorest quintile had the largest number of women (373).

**Table 1:** Prevalence of PIPV against women and problem drinking

**Variable**	**PIPV**			**Partner gets drunk**		
	**Freq (%)**	**Weighted total**^**a**^	**Chi.sq.p-value**	**Freq (%)**	**Weighted total**^**a**^	**Chi.sq.p-value**
**Age group**						
15**–**24	183 (42.9)	426		191 (39.9)	478	
25–34	311 (51.1)	610	0.003	378 (52.4)	720	<0.001
35–44	208 (50.2)	414	(0.02 trend)	225 (54.9)	411	(<0.001 trend)
45+	65 (44.7)	146		64 (51.1)	132	
**Education level**						
None	180 (48.2)	372	<0.001	242 (59.5)	408	
Primary	526 (52.5)	1002	(<0.001 trend)	522 (48.3)	1083	<0.001
Secondary	54 (30.3)	179		82 (39.8)	205	
Tertiary	8 (19.3)	43		14 (31.6)	45	
**Residence**						
Urban	76 (33.6)	227	<0.001	101 (39.6)	254	<0.001
Rural	692 (50.5)	1369		760 (51.1)	1487	
**Marital status**						
Married	487(48.0)	1016		547 (48.7)	1124	
Living together	129 (44.9)	286	0.02	155 (45.6)	341	
Widowed/divorced	40 (39.9)	100		60 (54.8)	109	0.005
Not living together	112 (57.8)	194		99 (59.3)	167	
**Unilaterally/jointly made**^**b**^						
0**–**1 decisions	187 (47.9)	392		196 (44.3)	443	
2–4 decisions	428 (47.0)	911	0.63	506 (49.5)	1021	0.09
**Region**						
Central	114 (38.7)	295		157 (44.0)	357	
Kampala	32 (29.8)	108	<0.001	36 (29.4)	124	
Eastern	210 (55.9)	375	184 (46.0)	400	<0.001	
Northern	171 (50.8)	336		248 (64.5)	385	
Western	241 (50.2)	482		235 (49.5)	475	
**Religion**						
Catholic	348 (50.3)	691		461 (60.5)	762	
Protestant	265 (50.9)	521	<0.001	308 (53.9)	572	<0.001
Muslim	58 (35.3)	163		24 (13.5)	179	
Other	97 (44.1)	221		67 (29.6)	228	
**Occupation**						
None	31 (30.8)	99		44 (34.2)	129	
Professional/clerical	14 (26.9)	53		17 (31.4)	54	
Sales	47 (37.4)	126		64 (43.7)	147	
Agriculture-self employed	559 (50.9)	1097	<0.001	608 (51.5)	1181	<0.001
Manual	47 (52.0)	90		50 (54.1)	92	
Others (agric/domestic/services)	69 (53.6)	131		73 (56.4)	138	
**Wealth status**						
Poorest	171 (54.0)	300		237 (67.5)	352	
Poorer	177 (56.0)	316		188 (50.4)	373	
Middle	165 (49.3)	334	<0.001	167 (50.6)	331	<0.001
Richer	155 (47.0)	329	(<0.001 trend)	150 (43.8)	342	
Richest	101 (31.7)	317		118 (34.5)	343	
**Education level of partner**						
None	76 (52.3)	146		116 (67.9)	170	
Primary	484 (51.4)	941		523 (50.7)	1032	<0.001
Secondary	156 (45.3)	344	<0.001	159 (43.6)	364	
Tertiary	34 (29.9)	113	(<0.001 trend)	39(33.0)	118	
Don’t Know/missing	18 (34.8)	52		25 (44.1)	57	
**Occupation of partner**						
Professional/clerical	47 (34.0)	137		56 (39.0)	145	
Sales	75 (43.6)	172	0.007	89 (46.8)	189	
Agriculture-self employed	431 (51.9)	829		481 (53.5)	900	<0.001
Manual	135 (45.1)	300		133 (41.3)	322	
Others (agric/domestic/services)	80 (50.9)	158		102 (55.2)	185	
**Age difference with partner**^**b**^						
Respondent Younger/same age	52 (49.2)	107		63 (52.4)	121	
1–4	231 (49.6)	466	0.28	246 (46.7)	526	0.29
5–9	193 (45.4)	426		216 (45.2)	478	
10+	139 (46.1)	301		175 (51.7)	339	
**All**	**768(48.1)**	**1596**		**861 (49.5)**	**1741**	

^a^PIPV *has special sample weights while drunkenness uses general sample weights.*

^*b*^*For only those who were married or were in a relationship at the time of the survey.*

The partners of the women were more educated and less engaged in agriculture or self employment compared to them. Only 9% (146) of the women reported that their partners were uneducated while 29% (457) said their partners attained at least secondary education. Fifty two percent of the women (829) said their partners were engaged in agriculture or were self employed.

Regarding age difference with their partners, 36% of the women reported that their partners were 1–4 years older than them, 33% being 5–9 years older and 23% 10 or more years older.

### Prevalence of drunkenness and PIPV

The overall prevalence of PIPV was 48%. PIPV varied significantly by age group of the women (p = 0.003), education level, (p < 0.001), residence (p < 0.001), marital status (p = 0.01), region (p < 0.001), religion (p < 0.001), occupation (p < 0.001), wealth status (p < 0.001), education level of the partner (p < 0.001) and occupation of the partner (p = 0.007) (Table 1). PIPV was most frequently reported among women aged 35–44 years, who did not have any formal education, resided in rural areas, did not live with their partners, lived in eastern region of the country, were Christians, engaged in manual labour, were the poorest, had partners who were engaged in agriculture/self employed and had partners who had not attained more than primary level of education. On average the older the women, the higher the likelihood of having experienced PIPV (p_trend_ = 0.02) and, the higher the education level of the women the less the likelihood of having experienced PIPV (p_trend_ < 0.001). Similarly, the higher the wealth status of the women the less the likelihood of having experienced PIPV (p_trend_ < 0.001). The prevalence of PIPV among the women did not significantly vary by participation in decision making and age difference with their partners.

Fifty six percent of the women reported that their partners drank alcohol. The proportion of women who reported that their partners got drunk was 49.5% and this represented 88% of those whose partners drank alcohol (56.0%). The levels and demographic patterns of PIPV were similar to those of problem drinking. Like PIPV the level of problem drinking significantly varied by age group (p < 0.001), educational level (p = 0.001), residence (p < 0.001), region (p < 0.001), religion (p < 0.001), occupation (p = 0.001), wealth status (p < 0.001), education level and occupation of partner (p < 0.001). The proportion of women who reported that their partners got drunk was lowest (42%) among those aged 15–24 but rose to 52% in next age group. The older the women the higher the likelihood of having a partner that got drunk (p_trend_ < 0.001). The higher the education level of a woman the lower the likelihood of having a partner who got drunk (p_trend_ < 0.001). Similarly, the wealthier the household of a woman the lower the likelihood of having a partner who got drunk. The problem drinking of the partners was most commonly reported by women from rural areas, from the western region, Catholics, those employed in manual labour, the poorest and those whose partners were in agriculture and self employed. The proportion of women whose partners got drunk did not significantly vary by participation in decision making and age difference with their partners.

### IPV and problem drinking

Table 2 shows results from bivariate and multivariate logistic regression analyses. The women whose partners drunk often were 6 times more likely to be victims of PIPV compared to those whose partners never drank alcohol (OR: 1.50-7.88). Those women whose partners got drunk only sometimes were 2.5 times more likely to be victims of PIPV compared to those whose partners never drank alcohol (OR: 1.99–3.15). There was no significant difference in likelihood of being a victim of PIPV between those whose partners never drank alcohol and those whose partners drank alcohol but did not get drunk. A chisquare test for trend of the proportion of the victims of PIPV by frequency of drunkenness of the partners was significant at 5% level (p_trend_ < 0.001).

**Table 2:** Unadjusted and adjusted Odds ratios for experience of PIPV by different characteristics

	**Unadjusted**		**Adjusted**^**1**^		**Wald’s test:**
**Variable**	**OR**	**95%CI**	**OR**	**95%CI**	**Chi-sq p-values**
**Problem drinking (base = never)**					
Drinks but doesn’t get drunk	*0.94*	*0.61–1.46*	*1.00*	*0.64–1.57*	*Chi-sq = 160 df = 3*
Sometimes	*2.50*	1.99–3.15***	*2.56*	2.01–3.26***	*<0.001*
Often	*6.00*	4.50–7.88***	*5.94*	4.43–7.97***	
**Age group (base = 15–24)**					
25**–**34	1.50	1.19–1.90**	1.32	1.03–1.70*	Chi-sq = 5 df = 3
35–44	1.53	1.17–2.00**	1.25	0.93–1.66	0.15
45+	1.28	0.87–1.88	1.06	0.70–1.60	
**Education level (base = none)**					
Primary	1.08	0.86–1.35			
Secondary	0.48	0.34–0.68***			
Tertiary	0.33	0.17–0.66**			
**Residence (Base = urban)**					
Rural	1.70	1.29–2.24***			
**Marital status (married)**					
Living together	0.75	0.59–0.96*			
Widowed/divorced	0.92	0.62–1.37			
Not living together	1.41	1.00–1.98*			
**Region (base Central)**					
Kampala	0.76	0.50–1.16	1.24	0.77–2.02	Chi-sq = 17 df = 4
Eastern	1.82	1.36–2.46***	1.73	1.25–2.41**	0.002
Northern	1.58	1.19–2.09**	1.03	0.73–1.45	
Western	1.70	1.26–2.29***	1.45	1.05–2.00*	
**Religion (base = Catholic)**					
Protestant	0.94	0.75–1.17			
Muslim	0.44	0.32–9.63***			
Other	0.61	0.45–0.82**			
**Occupation (base = none)**					
Professional/clerical	1.19	0.76–1.88			
Agriculture/self employed	2.07	1.43–3.01***			
Manual	2.45	1.49–4.02***			
Others	2.55	1.49–4.36**			
**Wealth status (base = poorest)**					
Poorer	0.89	0.67–1.18	1.11	0.81–1.51	Ch-sq = 12 df = 4
Middle	0.80	0.60–1.08	0.92	0.65–1.32	0.016
Richer	0.82	0.61–1.10	1.05	0.74–1.50	
Richest	0.41	0.30–0.55***	0.60	0.40–0.89*	
**Education level of partner (base = none)**					
Primary	1.08	0.86–1.34			
Secondary	0.48	0.34–0.68***			
Tertiary	0.33	0.17–0.66**			
**Occupation of partner (base = Prof/clerical)**					
Sales	1.51	0.96–2.36			
Agriculture-self employed	1.89	1.31–2.74**			
Manual	1.36	0.90–2.06			
Others (agric/domestic/services)	1.54	0.98–2.42			
**Fitness of model**					
% of observations correctly classified			65%		
Pearson’s Goodness of fit test p-value			0.72		

NB: When education replaced wealth status in final model it had chi.sq of 23 with a p < 0.001.

^1^Adjusted Odds ratios were derived from a final multivariate model. Several variables that were not significant were eliminated from the model.

The relationship between problem drinking of the women’s partner and likelihood of experiencing PIPV remained strong after controlling for age group, region and wealth status. In terms of contribution to the overall significance of the model problem drinking contributed most (p < 0.001) compared to age group (p = 0.15), wealth (p = 0.016) and region (p = 0.002). Education also contributed highly (p < 0.001) but had a lower chi-square value (23) compared with problem drinking (166) for the same degrees of freedom (3). Therefore, problem drinking was the strongest correlate of PIPV. The adjusted odds ratios for levels of problem drinking remained virtually the same. The model fitted well with Pearson’s chi-square test p-value of 0.72 which was greater than 0.05 expected of well fitting models [41]. Education attainment was also significant but was not included in the same model with wealth status because they were both highly correlated. Interaction terms for problem drinking and each of the significant variables were not significant (age group, p = 0.71; region, p = 0.95; wealth status, p = 0.15; and education, p = 0.20). This shows that the strength of the correlation between problem drinking and PIPV did not significantly vary by levels of age group, region, wealth status and education status of the women.

## Discussion

The results showed that the population prevalence rates of PIPV against women in Uganda and problem drinking of their partners are quite high (PIPV-48%, problem drinking-49.5%). The level of PIPV against women is higher than the levels reported for the USA (11.5% among whites, 23% blacks, and 17% Hispanic couples), Canada (7%) and Japan (13%) [17,29,38]. However, the findings concur with earlier surveys in Uganda [35,36]. It is not surprising that the level of PIPV against women in Uganda is nearly the same as that for Tanzania (47%) [38] because it is a neighbouring country with nearly similar cultural, economic and demographic make-up.

The findings about problem drinking, and particularly getting drunk, being the strongest predictor of PIPV among those factors investigated (and with potentially confounding influences controlled) are similar to those in previous studies in the UK and the USA [20,21]. The World Health Organization (WHO) already recognizes problem drinking as a determinant of PIPV and advises countries to reduce access and harmful use of alcohol [42]. Other studies have also strongly attributed PIPV to problem drinking with some showing that it contributes over 40% of the severe violence [17,43].

The findings indicate that PIPV may not have much relationship with the male partner’s simple consumption of alcohol or not but rather his pattern, his pattern of consumption, ever being drank and especially often being so, that is the occurrence and frequency of drunkenness. The proportion of men that drinks alcohol in Uganda is much lower than that of many developed countries but a considerable proportion of drinkers (almost nine out of ten) is reported by their spouses to be occasionally drunk. For example 88% of men take alcohol in the UK [44] as opposed to 55% of men in Uganda [32]. However, 59% of those who drink in UK get drunk [45] compared to 88% among the Ugandan men who drink alcohol. The level of PIPV in Uganda is also much higher than that in developed countries. The level of PIPV in UK is 28% while in Uganda it is 48% [34]. Peru, Ethiopia, Zambia, and Tanzania also have high levels of PIPV but they are among countries with lowest per capita alcohol consumption [31,46]. A country’s pattern of heavy drinking may not be predictive of likelihood of PIPV but in some countries such as Uganda it is highly predictive.

The high proportion of individuals who drink alcohol in UK, with relatively lower rates of PIPV is probably due to a lower proportion of men who get drunk among those who consume alcohol. Our findings show similar levels of PIPV among men who do not drink and those who drink but do not get drunk. This study has further shown that drinking that leads to obvious intoxication that a spouse might detect and report, can be dangerous. This indicates that governments can mitigate the PIPV problem by focusing more on reduction of problem drinking rather than completely stopping people from drinking, which would be a tougher task. This is also in line with WHO advice on reducing PIPV through moderating alcohol consumption [2]. Most of the Ugandan drinkers take alcohol in bars (69%) [32], which provides an opportunity for regulation of drinking hours and selling of alcohol to people who are visibly getting intoxicated. This can be difficult but there have been some successful interventions. A review of ten studies found that policies that reduced on-premises drinking hours by 2 h or more significantly reduced harmful use of alcohol [47]. During the reign of Idi Amin from 1971–1979 government reduced excessive drinking through banning of consumption of alcohol during working hours [48,49] but this was enforced by arrests and other means of terror that may not work in current modern society. However it shows that government intervention may have an impact. Further reduction in PIPV may also require addressing other concomitant factors such as women education and empowerment which are highlighted in these findings.

Most of the independent factors examined for a relationship with PIPV were found significant but none moderated the relationship between PIPV and problem drinking of the sexual partners of the women. The strength of the relation between problem drinking and PIPV did not even change by different levels of significant correlates of PIPV like age group, region, wealth status and education. This underlines the need for further studies to identify factors that might substantially modify the relationship between alcohol consumption and PIPV.

Findings of higher risk of PIPV among those with less education attainment, low wealth status are consistent with several studies [24-26]. Our descriptive results are also in line with findings from a crossnational analysis of PIPV in 40 countries which show that access to formal education and paid employment can create important gains in the worldwide efforts to reduce PIPV against women by promoting women’s self-determination [50].

While most of the findings in this study are consistent with some of the previous research done a few are inconsistent. PIPV was more common in rural areas and among older people but in research in other countries it was found more common in urban areas [25] and among younger people [26]. The lower levels of PIPV in Kampala (the capital) and the central region compared to other regions of Uganda can be explained by higher wealth status level and more education attainment in Kampala. The lower level of PIPV among Muslims and other denominations of Christians (mostly evangelicals) compared to Catholics and Protestants may be explained by the lower level of alcohol consumption among these religious groups.

Our study findings are subject to several limitations. The information on male partners’ drinking habits and PIPV was provided by the women, introducing the possibility of self-report data errors and misclassification biases. Classifying the frequency of drunkenness as often or sometimes was also subjective lacking specific frequency-in-time information. Nevertheless, we believe it to be a noteworthy strength of the study that women, who bear the largest burden of PIPV, and also experience the externalities associated with partners’ problem drinking, which they can observe, are the source of the reports.

Secondly, the period in which PIPV and problem drinking occurred may have involved different time periods depending on length of the relationship of the respondent and her partner. This brings with it potential for recall bias, which could in future be better addressed by adding queries of the timing between PIPV and problem drinking. Being based on a cross-sectional self-report survey, it is inherently difficult to tell whether PIPV started after problem drinking or it was the reverse, or that both occurred in approximately the same period. However, this study highlights associations between problem drinking and IPV as well as estimating the influences of women’s education, their empowerment, their partners’ education and their socio-economic status in mitigating this harm. These findings have importance for informing interventions for IPV in Uganda and elsewhere, and can help to generate hypotheses regarding PIPV and alcohol consumption to motivate and sharpen the focus of further research efforts.

## Conclusions

The prevalence of reported PIPV among women and problem drinking among their partners are relatively high in Uganda. There is a very strong association between experience of PIPV among women and problem drinking of their partners. The more often men get drunk the more they are likely to inflict PIPV against their spouses.

Besides problem drinking lower education attainment, low wealth status, higher age group and region are correlated with PIPV. Although results are cross-sectional and causality cannot be established, results are consistent with the notion that increasing education levels of women and their partners would have the potential to reduce exposure to PIPV. Similarly, increasing wealth or socio-economic status of women in general might reduce the risk of PIPV.

Among the strengths of this study are that the survey provided nationwide coverage and involved women respondents who are most affected by PIPV. The sample size was large enough to allow reliable prevalence estimation, and significance testing of demographic differences in prevalence of PIPV and drunkenness. Finally, analysis of multivariate models that predict PIPV and include problem drinking while controlling for other important factors brings out the importance of victimization, drunkenness, or problem drinking in Uganda, an important African country because of its high levels of both problem drinking and physical IPV.

More effort is required to reduce PIPV and problem drinking in Uganda. Freedom from violence is a basic human right, and policies to reduce physical intimate partner violence (PIPV) need to incorporate strategies to regulate heavy alcohol consumption among men as well as improve education and empowerment of women. Campaigns against PIPV should focus more on the older people, the less educated and the poor, as part of addressing regional economic and social development. Additional information for targeting PIPV prevention programs are suggested because of elevated levels seen among rural residents, Christians, and those employed in manual labour and peasant farming. Examples of related campaigns that have made a significant difference include the “raising voices” programme in Uganda which used discussions on use of power to discourage wife and child beating and the “we can” campaign of Oxfam in Asia which significantly increased awareness of gender equity and rejection of violence [51].

Future Demographic and Health surveys or other nationwide surveys should add additional questions and specific measures of heavy drinking, for example maximum amount consumed on any day and number of drinks to feel drunk, as well as of circumstances surrounding the intimate partner violence, so as to enable more detailed analysis of alcohol consumption patterns and their relationship with PIPV. More research should be carried out to identify evidence-informed interventions to mitigate problem drinking and PIPV.

Now that many studies have established an association between alcohol use and PIPV there is a need for meta-analysis and Cochrane reviews to provide more authoritative evidence on the relationship between the two.

## Competing interests

None identified. The author(s) declare that they have no competing interests.

## Authors’ contributions

All authors read and approved the final manuscript. NMT initiated the topic, analyzed the data and wrote the first draft of the paper. GKB and TKG made critical input and comments on the draft manuscript. RKW contributed to the interpretation and writing of the paper.

## Pre-publication history

The pre-publication history for this paper can be accessed here:

http://www.biomedcentral.com/1471-2458/12/399/prepub
